# *Bacillus megaterium* 1259 Limits Hepatic Lipid Accumulation in Mice with Established Diet-Induced Obesity: Paired Hepatic Transcriptomic and Metabolomic Profiling

**DOI:** 10.3390/nu18213687

**Published:** 2026-11-06

**Authors:** Yunfei An, Jiatian Yu, Xinyu Zhang, Ziyu Su, Meiyang Dong, Jingyi Wang, Zixuan Zhang, Tianyu Yang

**Affiliations:** 1 Experimental Conditions Support Center, Xuzhou Medical University, Xuzhou 221004, China; 100002024033@xzhmu.edu.cn (Y.A.);; 2 School of Basic Medicine and Life Sciences, Xuzhou Medical University, Xuzhou 221004, China; jiatianyu@xzhmu.edu.cn; 3 School of Medical Imaging, Xuzhou Medical University, Xuzhou 221004, China; 15873668022@163.com (X.Z.);; 4 School of First Clinical Medical, Xuzhou Medical University, Xuzhou 221004, China

**Keywords:** *Bacillus megaterium* 1259, diet-induced obesity, hepatic steatosis, microbial intervention, lipid metabolism, transcriptomics, metabolomics

## Abstract

Background: Obesity promotes ectopic lipid deposition and hepatic steatosis, a central feature of metabolic dysfunction-associated steatotic liver disease. Because microbial effects are strain- and model-specific, whether *Bacillus megaterium* 1259 (BM1259) affects established diet-induced obesity remains unclear. We evaluated BM1259 during continued high-fat diet (HFD) exposure and examined accompanying hepatic changes. Methods: Male C57BL/6J mice received an HFD for 12 weeks before assignment to HFD + PBS or HFD + BM1259 groups; normal-diet mice served as controls. BM1259 (1 × 10^10^ CFU/mouse/day) was administered by intragastric gavage for 8 weeks during continued HFD feeding. Phenotypic, biochemical, and histopathological assessments were combined with hepatic RNA sequencing, untargeted metabolomics, and paired multi-omics analysis. Results: Body weight was significantly lower in the HFD + BM1259 group at the intervention endpoint. BM1259 administration was also associated with lower body-weight-normalized iWAT and liver weights, a smaller mean iWAT adipocyte area, and lower hepatic triglyceride content and Oil Red O-positive area. Serum LDL-C, ALT, and fasting blood glucose were lower, whereas the reduction in ipGTT AUC did not reach statistical significance. Hepatic transcriptomic differences involved genes related to lipid uptake, synthesis, lipoprotein handling, and fatty-acyl processing. Metabolomic differences included features related to choline–glycerophospholipid, sphingolipid, steroid-related, and acylcarnitine metabolism. Exploratory paired analysis suggested possible associations between gene expression and metabolite abundance. Conclusions: BM1259 was associated with lower obesity-related and hepatic lipid burden during continued HFD exposure and warrants further investigation as a candidate microbial intervention.

## 1. Introduction

Obesity is accompanied by progressive changes in adipose-tissue function, not simply an increase in fat mass. Once storage capacity becomes limited, adipocyte hypertrophy and impaired tissue expandability favor lipid overflow into non-adipose organs, particularly the liver [1,2]. Hepatic steatosis is a defining feature of metabolic dysfunction-associated steatotic liver disease (MASLD), the current term for fatty liver disease occurring in the presence of cardiometabolic risk factors [3,4]. MASLD can progress from isolated steatosis to steatohepatitis, fibrosis, cirrhosis, and hepatocellular carcinoma, and its global burden has risen in parallel with obesity and type 2 diabetes [4,5,6]. Because adipose-tissue dysfunction, insulin resistance, dyslipidemia, and hepatic lipid deposition commonly coexist, an intervention tested after obesity has developed addresses a different question from one introduced at the start of high-fat feeding [1,4,5,6,7].

Hepatic steatosis reflects an imbalance among lipid acquisition, synthesis, oxidation, storage, and export. Fatty acids reach the liver from adipose-tissue lipolysis and dietary sources, while de novo lipogenesis provides an additional intrahepatic contribution; disposal depends mainly on fatty-acid oxidation and secretion in very-low-density lipoproteins [7,8,9,10]. Prolonged disruption of these processes expands hepatic triglyceride stores and alters bioactive lipid species involved in metabolic signaling and cellular stress [8,10,11]. The amount of stored triglyceride, however, does not alone determine liver injury. Lipid composition and intracellular distribution, together with mitochondrial adaptation, oxidative stress, and inflammation, influence whether steatosis progresses toward lipotoxic damage [8,10,11,12,13]. Beyond bulk triglyceride storage, hepatic glycerophospholipid and sphingolipid composition provides additional information on lipoprotein handling, metabolic signaling, and lipotoxic stress [9,11,13]. Evaluating tissue pathology together with hepatic transcriptional and metabolic profiles can therefore distinguish changes in lipid burden from the broader molecular adjustments that accompany an intervention.

Defined microbial preparations are being investigated as metabolic interventions, but their effects cannot be treated as a class property. Microorganisms and their products can interact with dietary substrates, bile acids, endocrine signals, and host immune and metabolic pathways [14,15]. At the same time, obesity-associated microbial signatures vary among human studies, and many observed differences remain difficult to classify as causes rather than consequences or correlates of disease [14,15,16,17]. Meta-analyses of probiotic supplementation have found modest and heterogeneous changes in body weight and selected metabolic outcomes, with responses differing by strain, dose, population, and treatment duration [18,19]. Human proof-of-concept studies with *Akkermansia muciniphila* and *Bifidobacterium longum*, together with experiments using several defined bacterial strains in diet-induced obesity models, further show that metabolic responses are neither uniform across organisms nor consistent across measured outcomes [20,21,22,23,24,25,26,27,28]. Strain identity, host model, disease stage, and intervention schedule must therefore be considered when evaluating a microbial candidate.

The same strain-specific standard applies to spore-forming *Bacillus* species. The accepted definition of a probiotic requires a demonstrated health benefit for the specific strain and intended use [29,30]. Although *Bacillus* spores may offer practical stability and tolerate gastrointestinal conditions, safety and biological activity must still be established individually [31]. Genomic and in vitro studies of selected *Bacillus megaterium* strains have identified digestive, antimicrobial, antioxidant, or metabolic properties, but these observations do not establish comparable effects in vivo [32]. BM1259, originally isolated from chicken manure, carries genes related to nutrient and nitrogen metabolism [33]. Studies in dairy cows and Holstein bull calves reported changes in ruminal fermentation, nutrient utilization, and production-related measures without evident adverse alterations in the blood biochemical variables examined [33,34]. Whether BM1259 modifies obesity-related phenotypes after obesity is established remains unknown. We therefore administered BM1259 to male C57BL/6J mice after 12 weeks of high-fat feeding while maintaining the dietary challenge for a further 8 weeks. Obesity-related phenotypes and liver pathology were assessed together with paired hepatic transcriptomic and untargeted metabolomic analyses to characterize the changes accompanying BM1259 administration and define priorities for subsequent mechanistic testing.

## 2. Materials and Methods

### 2.1. Preparation of Bacillus megaterium 1259

*Bacillus megaterium* 1259, originally isolated from chicken manure in Yangzhou, China, was deposited in the China General Microbiological Culture Collection Center (CGMCC No. 1259). Its taxonomic identification by 16S rRNA gene sequencing and phylogenetic analysis has been reported previously, and the corresponding genome-annotation study reported that no pathogenic genes were identified [33]. BM1259 was obtained from Yangzhou University (Yangzhou, China) and cultured in Luria–Bertani medium at 37 °C. Cells were harvested by centrifugation at 12,000 rpm for 10 min at 4 °C, washed three times with phosphate-buffered saline (PBS), and resuspended in PBS. A fresh suspension providing 1 × 10^10^ colony-forming units (CFU) per mouse was prepared daily for intragastric gavage. The viable concentration of the final PBS suspension was determined by serial dilution and plate counting before gavage.

### 2.2. Animals and Experimental Design

A total of 24 seven-week-old male specific-pathogen-free C57BL/6J mice were obtained from the Laboratory Animal Center of Xuzhou Medical University (Xuzhou, China). Mice were housed four per cage at 22 ± 1 °C and 30–70% relative humidity under a 12 h light/12 h dark cycle, with unrestricted access to food and water. After a 1-week acclimation period, mice were fed either a normal diet (ND; 10% of energy from fat; catalog no. XT10LZJ-009) or a high-fat diet (HFD; 60% of energy from fat; catalog no. XTHF60). Both diets were supplied by Xietong Biotechnology (Nanjing, China).

After 12 weeks of HFD feeding, mice whose body weight was at least 20% greater than the mean body weight of age-matched ND-fed mice were considered to have diet-induced obesity. Eligible HFD-fed mice were stratified by body weight and randomly assigned to the HFD + PBS or HFD + BM1259 group. Age-matched ND-fed mice constituted the ND + PBS group. Each group initially consisted of eight mice to ensure that at least six independent biological replicates per group would be available for endpoint phenotypic assessments. All eight mice per group were monitored for body weight. Before the endpoint assessments, six mice per group were randomly selected for the respective phenotypic analyses, and four of these six mice in each HFD-fed group were randomly selected for paired RNA-seq and metabolomic profiling.

During the subsequent 8-week intervention, mice remained on their assigned diets. The HFD + BM1259 group received BM1259 at 1 × 10^10^ CFU/mouse once daily by intragastric gavage, whereas the ND + PBS and HFD + PBS groups received an equal volume of PBS. Body weight and food intake were measured twice weekly at a fixed time of day and food intake was measured at the cage level. For each cage, food consumption during each measurement interval was calculated by subtracting the amount of food remaining and spilled from the amount provided. The corrected food consumption was then divided by the number of mice in the cage and the number of days in the measurement interval and expressed as g/mouse/day. Fasting blood glucose, intraperitoneal glucose tolerance, serum biochemical indices, tissue weights, hepatic triglyceride content, and histopathology were assessed during intervention week 8.

At the endpoint, blood was collected from the retro-orbital venous plexus before euthanasia by carbon dioxide inhalation. Liver and adipose tissues were excised and weighed. Tissue samples were either fixed in 4% paraformaldehyde or snap-frozen in liquid nitrogen and stored at −80 °C. All procedures were approved by the Animal Ethics Committee of Xuzhou Medical University (approval no. 202509T015).

### 2.3. Intraperitoneal Glucose Tolerance Test

The ipGTT was performed during intervention week 8 after a 6 h fast, with water available ad libitum. Tail-vein blood glucose was measured immediately before the intraperitoneal administration of D-glucose at 1.5 g/kg body weight. The glucose dose was calculated using the body weight recorded on the day of testing. Blood glucose was subsequently measured at 15, 30, 60, 90, and 120 min using a Safe AQ Pro III glucometer and compatible test strips (Sinocare, Changsha, China). The area under the glucose concentration–time curve was calculated using the trapezoidal rule.

### 2.4. Fasting Blood Glucose, Serum Biochemistry, and Hepatic Triglyceride Measurement

Fasting blood glucose was measured during intervention week 8 after a 6 h fast using tail-vein blood and a Safe AQ Pro III glucometer (Sinocare, Changsha, China). Hepatic triglyceride (TG) content was measured in liver tissue using a commercial triglyceride assay kit (catalog no. A110-1-1; Nanjing Jiancheng Bioengineering Institute, Nanjing, China). Liver tissue homogenates were prepared and analyzed according to the manufacturer’s instructions. TG content was determined using the GPO-PAP enzymatic colorimetric method and normalized to wet tissue mass.

Terminal blood samples were allowed to clot at room temperature for 1 h and were centrifuged at 3000 rpm for 10 min at 4 °C. Serum was aliquoted, stored at −20 °C, and protected from repeated freeze–thaw cycles. Total cholesterol, triglycerides, high-density lipoprotein cholesterol, low-density lipoprotein cholesterol, alanine aminotransferase, and aspartate aminotransferase were measured at the Affiliated Hospital of Xuzhou Medical University (Xuzhou, China) using a fully automated clinical chemistry analyzer according to the clinical laboratory’s standardized procedures.

### 2.5. Histopathological Analysis

Inguinal and epididymal white adipose tissues and liver samples were fixed in 4% paraformaldehyde for 24 h, dehydrated, embedded in paraffin, and sectioned at a thickness of 5 μm. After deparaffinization and rehydration, the sections were stained with hematoxylin and eosin (H&E). H&E-stained images of adipose tissue were acquired at 20× magnification. Adipocyte cross-sectional area was quantified using Fiji/ImageJ version 2.18.0/1.54p (National Institutes of Health, Bethesda, MD, USA) with identical segmentation settings across all images. Non-overlapping fields were analyzed per mouse. Artifacts and incomplete adipocytes intersecting the image boundary were excluded. Measurements from all eligible adipocytes were averaged to obtain one mean adipocyte-area value for each mouse.

For Oil Red O staining, fixed liver samples were embedded in optimal cutting temperature compound and cryosectioned at a thickness of 5 μm. The sections were stained with Oil Red O to visualize neutral lipids and counterstained with hematoxylin. Oil Red O-stained liver sections were imaged at 20× magnification, and non-overlapping tissue fields were exported as TIFF files. Images were analyzed using Fiji/ImageJ. Oil Red O-positive pixels were segmented in the HSB color space using identical fixed threshold settings for all images. The Oil Red O-positive area was expressed as a percentage of the total analyzed tissue area. Measurements from multiple fields were averaged to obtain one value for each mouse.

### 2.6. Liver Transcriptome Sequencing and Bioinformatic Analysis

Hepatic transcriptomic profiling was performed using four randomly selected, biologically independent mice from each HFD-fed group. Total RNA was extracted from frozen liver tissue using QIAzol Lysis Reagent (Qiagen, Hilden, Germany). RNA quality was assessed using a 5300 Bioanalyzer (Agilent Technologies, Santa Clara, CA, USA), and RNA concentration was measured using an ND-2000 spectrophotometer (NanoDrop Technologies, Wilmington, DE, USA). Strand-specific mRNA libraries were prepared from qualified RNA and sequenced as 150 bp paired-end reads on a DNBSEQ-T7 platform by Shanghai Majorbio Bio-pharm Technology Co., Ltd. (Shanghai, China).

Raw reads were quality-filtered using fastp version 1.4.0 and aligned to the *Mus musculus* reference genome using HISAT2 version 2.2.1. Transcripts were assembled using StringTie version 2.2.3, and gene abundance was estimated using RSEM version 1.3.3. Transcripts-per-million values were used for principal component analysis and expression visualization, whereas raw counts were used for differential-expression analysis with DESeq2 version 1.52.0. Genes with an adjusted *p* value < 0.05 were considered differentially expressed. Gene Ontology enrichment was performed using GOATOOLS version 1.6.9, and Kyoto Encyclopedia of Genes and Genomes pathway enrichment was assessed using Fisher’s exact test implemented in SciPy version 1.17.0. An adjusted *p* value < 0.05 was considered statistically significant. Analyses were conducted on the Majorbio Cloud Platform(Majorbio, Shanghai, China).

### 2.7. Quantitative Real-Time Polymerase Chain Reaction Analysis (RT-qPCR)

Total RNA was extracted from liver tissue using RLT Lysis Reagent (NCMBIO, Suzhou, China). Total RNA was reverse-transcribed into complementary DNA using HisyGo RT Red SuperMix for qPCR (+gDNA Wiper) (Vazyme, Nanjing, China). Quantitative real-time PCR was performed using SYBR Green I intercalating-dye chemistry on a LightCycler 480 II real-time PCR system (Roche Diagnostics, Basel, Switzerland). *Gapdh* was used as the reference gene, and relative mRNA expression was calculated using the 2^−ΔΔCt^ method, with the HFD + PBS group used as the calibrator. The primer sequences are shown in Table S1.

### 2.8. Liver Untargeted Metabolomics

Untargeted metabolomic profiling was performed using the same liver samples analyzed by transcriptomics, with four mice included from each HFD-fed group. Approximately 50 ± 5 mg of frozen liver tissue was extracted with 400 μL of methanol/water (4:1, *v*/*v*) containing internal standards. Samples were homogenized, sonicated, incubated at −20 °C, and centrifuged at 13,000× *g* for 15 min at 4 °C. The resulting supernatants were collected for analysis. A pooled quality-control sample was prepared by combining equal volumes of all supernatants and was injected periodically to monitor analytical stability.

Metabolites were analyzed using a Vanquish ultra-high-performance liquid chromatography system coupled to an Orbitrap Exploris 120 mass spectrometer (Thermo Fisher Scientific, Waltham, MA, USA). Chromatographic separation was performed using an ACQUITY UPLC HSS T3 column (100 mm × 2.1 mm, 1.8 μm; Waters, Milford, MA, USA). Mass spectra were acquired in positive- and negative-ion modes over an m/z range of 70–1050.

Raw data were processed using Progenesis QI version 3.0 for peak detection, alignment, and integration. Metabolites were putatively annotated by matching precursor masses and tandem mass spectra against the Human Metabolome Database, METLIN, and the Majorbio in-house database. Features with a relative standard deviation > 30% in the pooled quality-control samples were excluded. Principal component analysis and orthogonal partial least-squares discriminant analysis were conducted using the R package ropls version 1.6.2. Candidate differential metabolites were defined by a variable importance in projection score > 1 and a two-sided Student’s *t*-test *p* value < 0.05. Benjamini–Hochberg-adjusted *p* values were also calculated to evaluate the robustness of metabolite-level differences. Kyoto Encyclopedia of Genes and Genomes pathway enrichment was evaluated using Fisher’s exact test, and both unadjusted and Benjamini–Hochberg-adjusted *p* values were reported.

### 2.9. Integrated Transcriptomic and Metabolomic Analysis

Integrated analysis was based on the eight paired liver samples from the HFD + PBS and HFD + BM1259 groups, with four mice included per group. RSEM-derived transcripts-per-million values and processed metabolite intensities were matched using individual-animal identifiers. Genes and metabolites were selected from the differential analyses and pathway-enrichment results according to their relevance to the metabolic processes examined.

Pairwise Spearman correlation coefficients and two-sided nominal *p* values were calculated across the matched samples using Python version 3.9.19 and SciPy version 1.17.0. The resulting correlation matrix was visualized as a heatmap. No multiple-testing correction was applied to the correlations; accordingly, this analysis was considered exploratory.

### 2.10. Statistical Analysis

Phenotypic data were analyzed using GraphPad Prism version 10.1.2 (GraphPad Software, Boston, MA, USA). The individual mouse was the biological unit for endpoint measurements and the ipGTT. Serial data are presented as the mean ± SD. For endpoint measurements, individual data points are shown together with the median, interquartile range, and range, as specified in the figure legends. All statistical tests were two-sided.

Variables measured once across the three groups, including endpoint body weight, fasting blood glucose, tissue weights, serum biochemical indices, hepatic triglyceride content, ipGTT area under the curve, mean adipocyte area and Oil Red O-positive area, were analyzed using one-way analysis of variance followed by Tukey’s multiple-comparisons test. Serial glucose concentrations obtained during the ipGTT were analyzed using two-way repeated-measures analysis of variance, with group and time as factors, followed by Tukey’s multiple-comparisons test. RT-qPCR data were analyzed using an unpaired, two-tailed Student’s *t*-test. Omics-specific statistical procedures and multiple-testing corrections are described in the corresponding subsections. Statistical significance was defined as *p* < 0.05. Exact sample sizes are reported in the figure legends.

## 3. Results

### 3.1. BM1259 Was Associated with Lower Adipose and Hepatic Lipid Burden During Continued HFD Feeding

After 12 weeks of HFD feeding, eligible mice with diet-induced obesity were allocated to receive PBS or BM1259 for a further 8 weeks while HFD feeding continued. The experimental design is summarized in Figure 1. The mean body weights of the two HFD-fed groups were similar at allocation. Their mean trajectories subsequently diverged and body weight was significantly lower in the HFD + BM1259 group at the intervention endpoint (Figure 2A). Recorded cage-level food intake followed broadly similar descriptive trajectories in the HFD + PBS and HFD + BM1259 groups (Figure 2B).

The glycemic response was less uniform. During the intraperitoneal glucose tolerance test (ipGTT), glucose concentrations in the HFD + BM1259 group were lower during the late phase of the test, including at 120 min. The corresponding area under the curve was numerically lower than that in the HFD + PBS group, but the difference did not reach statistical significance (*p* = 0.073; Figure 2C). In contrast, fasting blood glucose was significantly lower following BM1259 administration, although it remained higher than that in the ND + PBS group (Figure 2D). These results support a reduction in fasting glycemia but not a conclusive improvement in overall glucose tolerance.

Body-weight-normalized inguinal white adipose tissue (iWAT) and liver weights were significantly lower in the HFD + BM1259 group than in the HFD + PBS group (Figure 2E,F). BM1259 administration was also associated with lower serum alanine aminotransferase and low-density lipoprotein cholesterol concentrations (Figure 2G,H). Hepatic triglyceride content was significantly lower in the HFD + BM1259 group than in the HFD + PBS group (Figure 2I). Representative iWAT sections showed less adipocyte hypertrophy, and quantitative histomorphometry confirmed a smaller mean adipocyte area (Figure 2J,K). Liver H&E and Oil Red O sections showed less extensive lipid accumulation, consistent with the significantly lower hepatic Oil Red O-positive area (Figure 2J,L). Body-weight-normalized epididymal adipose tissue (eWAT) and serum total cholesterol, triglyceride, high-density lipoprotein cholesterol, and aspartate aminotransferase did not differ significantly between the two HFD-fed groups (Figure S1). Overall, the phenotypic response was most evident in body weight, adipose-tissue burden, and hepatic lipid accumulation, with the hepatic phenotype supported by concordant biochemical and histological measurements, whereas the improvement in glucose regulation was partial.

### 3.2. BM1259 Was Associated with Coordinated Changes in Hepatic Lipid-Handling Transcriptional Profiles

Hepatic transcriptomes from the HFD + PBS and HFD + BM1259 groups were compared to characterize the liver transcriptional profile accompanying BM1259 administration. Principal component 1 and principal component 2 explained 39.30% and 20.88% of the total variance, respectively. Samples showed partial separation by treatment, although overlap between groups and within-group variability remained evident (Figure 3A). Differential-expression analysis identified 69 genes, comprising 26 upregulated and 43 downregulated genes in the HFD + BM1259 group at an adjusted *p* value < 0.05 (Figure 3B). Expression patterns for all 69 differentially expressed genes are presented in Figure S2, and complete gene-level statistics are provided in Table S2.

The focused heatmap showed that the selected lipid-related genes did not change uniformly. Hepatic transcript abundance of *Cd36*, *Vldlr*, *Apoa4*, *Me1*, *Acot2*, and *Dbi*, which are related to lipid uptake, lipoprotein transport, lipid synthesis, or fatty-acyl handling, was lower in the HFD + BM1259 group. In contrast, *Cyp7b1* and *Gpcpd1*, associated with steroid and glycerophosphocholine metabolism, respectively, showed higher transcript abundance (Figure 3C). These measurements establish differences at the transcript level. In addition, hepatic *Cd36* and *Me1* mRNA abundance was lower, whereas *Cyp7b1* and *Gpcpd1* mRNA abundance was higher in the HFD + BM1259 group than in the HFD + PBS group (Figure 3G). These results corroborated the selected differences at the mRNA level.

KEGG enrichment analysis identified retinol metabolism, glutathione metabolism, xenobiotic metabolism by cytochrome P450, steroid hormone biosynthesis, arachidonic acid metabolism, and PPAR signaling at adjusted *p* values < 0.05 (Figure 3D). The PPAR signaling annotation included the downregulated genes *Cd36*, *Me1*, and *Dbi*. Genes mapped to glutathione and xenobiotic metabolism included predominantly lower transcript abundance of members of the Gsta and Gstm families, together with *Mgst3*, *Gclc*, and *Ugt1a9*. Cytochrome P450 genes assigned to steroid and arachidonic acid metabolism changed in different directions. The enrichment results therefore reflected mixed transcriptional changes among pathway members rather than uniform pathway activation or suppression. To determine whether coordinated transcriptional differences extended beyond the differentially expressed genes, gene set enrichment analysis was performed using the complete ranked hepatic transcriptome. Reactome gene sets related to fatty acid metabolism, peroxisomal lipid metabolism, fatty acyl-CoA biosynthesis, plasma lipoprotein remodeling, triglyceride metabolism, and glycerophospholipid biosynthesis were enriched toward the HFD + PBS group (NES, −2.094 to −1.627; adjusted *p* < 0.05; Figure 3E,F). This pattern indicates relatively lower ranked expression of these lipid-related gene sets in the HFD + BM1259 group. Complementary Gene Ontology enrichment results are shown in Figure S3, and the complete Gene Ontology, KEGG and reactome GSEA results are provided in Table S3.

### 3.3. BM1259 Was Associated with Differences in the Hepatic Metabolic Profile and Lipid-Related Pathways

Untargeted metabolomics was used to compare hepatic metabolite profiles between the HFD + PBS and HFD + BM1259 groups. Principal component 1 and principal component 2 explained 40.50% and 17.20% of the total variance, respectively, with partial group separation occurring primarily along principal component 1 (Figure 4A). Pooled quality-control samples clustered closely, supporting the analytical reproducibility of the dataset.

Using a variable importance in projection score > 1 and a two-sided Student’s *t*-test *p* value < 0.05, 255 putatively annotated candidate differential metabolites were identified. Of these, 145 showed higher and 110 showed lower relative abundance in the HFD + BM1259 group. Thirteen candidates remained significant after Benjamini–Hochberg correction (Figure 4B). Hierarchical clustering further illustrated the relative-abundance patterns of the candidate metabolites across individual liver samples (Figure S4). Complete feature-level statistics, including fold change, variable importance in projection score, unadjusted *p* value, FDR, and annotation confidence, are provided in Table S4.

The six metabolites selected for individual display represented several components of the metabolomic profile. Allopregnanolone and Oct-5-Enedioylcarnitine showed higher relative abundance, whereas choline glycerophosphate, 1-stearoyl-2-hydroxy-PC [LPC(18:0)], dihydroceramide, and sphingosine showed lower relative abundance in the HFD + BM1259 group (all *p* < 0.01; Figure 4C). Allopregnanolone, Oct-5-Enedioylcarnitine, and choline glycerophosphate also retained FDR < 0.05. LPC(18:0), dihydroceramide, and sphingosine did not remain significant after multiple-testing correction. These candidates represented steroid- and acylcarnitine-related features together with choline–glycerophospholipid and sphingolipid species.

Of the candidate differential metabolites, 37 mapped to KEGG pathways. “Choline metabolism in cancer” (the official KEGG pathway name) and the sphingolipid signaling pathway remained significantly enriched after multiple-testing correction at adjusted *p* values < 0.05 (Figure 4D). Riboflavin metabolism, biosynthesis of cofactors, pyrimidine metabolism, and vitamin digestion and absorption also met the adjusted significance threshold. In contrast, glycerophospholipid metabolism, ether lipid metabolism, and sphingolipid metabolism were nominally enriched at unadjusted *p* values < 0.05 but did not remain significant after correction. In this context, “Choline metabolism in cancer” denotes shared choline- and phosphatidylcholine-related metabolic nodes in the KEGG database and does not indicate a cancer-related process. The complete pathway-enrichment results, including unadjusted and Benjamini–Hochberg-adjusted *p* values, are provided in Table S5.

### 3.4. Exploratory Integrated Analysis Identified Potential Hepatic Gene–Metabolite Associations

Spearman correlations were calculated across eight matched liver samples to examine covariation between selected transcriptomic and metabolomic features. Hierarchical clustering produced two broadly opposing association patterns (Figure 5A). *Gpcpd1* and *Cyp7b1* clustered together and showed positive associations with allopregnanolone but inverse associations with several choline–glycerophospholipid and sphingolipid metabolites. A cluster comprising *Apoa4*, *Me1*, *Dbi*, *Cd36*, and *Gclc* generally showed the opposite pattern.

Representative correlations included *Gclc* with choline glycerophosphate (Spearman’s ρ = 0.976), *Me1* with LPC(18:0) (ρ = 0.952), *Dbi* with LPC(18:0) (ρ = 0.905), and *Gpcpd1* with allopregnanolone (ρ = 0.881; all *p* < 0.01). Complete coefficients and nominal *p* values are provided in Table S6. However, the analysis included 49 comparisons without multiple-testing correction, and the pooled treatment-group structure could account for part or all of the apparent covariation. These patterns were therefore treated as descriptive and hypothesis-generating; no individual gene–metabolite pair was considered independently validated, and no within-group relationship was inferred.

Figure 5B presents a descriptive summary of the group-level phenotypic, transcript-level, and metabolite-level differences observed in the study rather than a validated mechanistic pathway.

## 4. Discussion

The clearest phenotypic finding was that BM1259 administration was accompanied by lower body-weight-normalized iWAT and liver weights, a smaller mean iWAT adipocyte area, lower hepatic triglyceride content and Oil Red O-positive area, and lower circulating LDL-C and ALT despite continued HFD feeding. The agreement between biochemical hepatic triglyceride measurement and Oil Red O morphometry strengthens the evidence for a lower hepatic lipid burden, although these measurements do not identify the metabolic process responsible for this difference. The lower body-weight trajectory during the later intervention period was directionally consistent with these endpoints. Mean recorded food intake showed similar patterns in the two HFD-fed groups, but measurements from only two cages per group do not establish equivalent caloric intake. The contribution of altered energy balance to the lower body weight and hepatic lipid burden therefore remains unresolved. Obesity-related liver injury develops from more than excess fat mass: impaired adipose-tissue storage, adipocyte hypertrophy, insulin resistance, and inflammatory and endocrine disturbances can increase lipid delivery to the liver and promote MASLD [3,4,35,36,37,38,39]. The phenotypic observations in this study therefore form a coherent pattern of lower adipose and hepatic burden. The accompanying molecular profiles were concentrated in hepatic lipid handling. Changes in lipid-related transcripts and choline–glycerophospholipid-related metabolites were prominent in their respective datasets; sphingolipid, steroid-related, acylcarnitine, and cofactor findings were supportive but less definitive. Their parallel occurrence supports prioritization for further investigation but does not establish functional coupling or a shared causal pathway.

The intervention schedule is central to the interpretation of these findings. BM1259 was introduced only after 12 weeks of HFD feeding, and the dietary challenge continued throughout the subsequent 8 weeks. The experiment therefore asked whether an established obese phenotype remained modifiable under ongoing nutritional pressure, rather than whether BM1259 could prevent obesity from developing. Defined bacterial preparations have reduced adiposity, dyslipidemia, or hepatic lipid accumulation in other HFD-fed mouse models, including studies of *Lacticaseibacillus paracasei* N1115, *Lactiplantibacillus plantarum* FRT4 and HF02, and human-derived *Faecalibacterium prausnitzii* strains [26,40,41,42]. These findings provide context but are not directly transferable to BM1259 because the organisms, doses, diets, and intervention schedules differ, and microbial effects are strongly strain- and setting-dependent [43]. Similarly, the characterization of *B. megaterium* MIT411 concerned mainly in vitro properties relevant to gastrointestinal use and did not establish an anti-obesity effect in mice [32]. The current data are therefore specific to BM1259 and this post-obesity design. They also show that the glycemic response was limited: fasting blood glucose was lower, but the decrease in ipGTT AUC was not statistically significant. The study supports lower fasting glycemia, not a definitive improvement in systemic glucose tolerance.

The liver transcriptome provides a molecular context for the hepatic phenotype. Transcript abundance of *Cd36*, *Vldlr*, *Apoa4*, *Me1*, *Dbi*, and *Acot2* was lower in the HFD + BM1259 group. Rather than defining a single linear pathway, these genes represent several aspects of lipid handling, including fatty-acid uptake, lipoprotein transport, NADPH supply, and intracellular fatty-acyl processing. *CD36* is relevant because it facilitates hepatic fatty-acid uptake and can promote SREBP1-dependent lipogenesis; hepatocyte-specific disruption of *Cd36* has reduced HFD-induced steatosis and insulin resistance in mice [44]. *ME1* converts malate to pyruvate while generating NADPH for fatty-acid and cholesterol synthesis, and loss of *ME1* has been associated with lower adiposity and hepatic steatosis during obesogenic feeding [45]. Lower *Vldlr*, *Apoa4*, *Dbi*, and *Acot2* transcript abundance extended the pattern to lipoprotein and acyl-group handling. RT-qPCR reproduced the direction of the RNA-seq differences for *Cd36*, *Me1*, *Cyp7b1*, and *Gpcpd1*, providing transcript-level corroboration for these selected genes. Whole-transcriptome GSEA extended these gene-level observations by showing that several Reactome gene sets related to fatty acid, peroxisomal lipid, fatty acyl-CoA, lipoprotein, triglyceride, and glycerophospholipid metabolism were enriched toward the HFD + PBS group. These results support a coordinated transcriptional difference in hepatic lipid handling but do not indicate uniform suppression of pathway activity by BM1259. In combination, these changes are consistent with reduced transcriptional representation of several lipid-acquisition and anabolic processes described in current models of hepatic lipid accumulation [9,13,38].

Choline–glycerophospholipid metabolism provided the strongest link between the transcriptomic and metabolomic datasets. Hepatic *Gpcpd1* transcript abundance was higher, whereas choline glycerophosphate and LPC(18:0) were lower. The KEGG pathway officially named “Choline metabolism in cancer” remained significant after multiple-testing correction, while glycerophospholipid metabolism was only nominally enriched and did not meet the FDR threshold. In this dataset, the former pathway name denotes shared choline- and phosphatidylcholine-related nodes and has no implication of a cancer process. *Gpcpd1* catalyzes the hydrolysis of glycerophosphocholine to choline and glycerol-3-phosphate, although the metabolic consequences of this reaction vary by tissue and physiological setting [46,47]. This reaction is relevant to hepatic lipid handling because phosphatidylcholine and its lysophospholipid derivatives contribute to membrane organization, lipid-droplet surfaces, and lipoprotein assembly [9,10,11]. Experimental disruption of phospholipid remodeling can also alter hepatic LPC(18:0) abundance and diet-induced steatosis [48]. The combination observed here supports altered glycerophosphocholine turnover, but it does not show that *Gpcpd1* activity or pathway flux increased. Targeted phospholipid measurements and isotope tracing will be required to distinguish changes in phosphatidylcholine synthesis, degradation, reacylation, and export and to determine whether any of these processes contributed to the hepatic phenotype.

The sphingolipid results describe a second, less resolved component of hepatic lipid remodeling. Dihydroceramide and sphingosine were lower at the nominal metabolite-selection threshold, although neither remained significant after FDR correction, whereas the sphingolipid signaling pathway remained enriched after multiple-testing adjustment. Ceramides and related sphingolipids participate in insulin signaling, mitochondrial function, oxidative stress, lipid storage, and hepatocyte injury, but their biological effects depend on molecular species, acyl-chain length, intracellular localization, and tissue context [11,49]. Experimental inhibition of dihydroceramide desaturase has improved insulin resistance and hepatic steatosis in obese mice [50], whereas hepatic sphingomyelin synthase 1 deficiency caused glucosylceramide accumulation and progressive liver pathology [51]. These contrasting outcomes illustrate why lower abundance of two annotated metabolites cannot be equated with suppression of ceramide synthesis or a uniform reduction in sphingolipid toxicity. A targeted panel of ceramides, dihydroceramides, sphingomyelins, and glycosphingolipids is needed to establish which species and synthetic routes were altered.

Steroid-related, acylcarnitine, and cofactor changes broadened the metabolomic response but did not provide a single explanatory mechanism. Allopregnanolone increased alongside higher *Cyp7b1* transcript abundance. *Cyp7b1* participates in oxysterol 7α-hydroxylation and the alternative bile-acid synthesis pathway [52,53], whereas allopregnanolone is a progesterone-derived neuroactive steroid that undergoes extensive hepatic metabolism [54]. Their concurrent changes justify examining steroid- and oxysterol-related metabolism but do not establish a direct *Cyp7b1*–allopregnanolone relationship. Oct-5-Enedioylcarnitine also increased. Acylcarnitines may reflect substrate entry, incomplete fatty-acid oxidation, peroxisomal or mitochondrial acyl-group handling, or metabolite export [55,56]; its increase therefore indicates altered fatty-acid handling rather than enhanced β-oxidation. Riboflavin metabolism and cofactor biosynthesis were also enriched. Riboflavin deficiency can aggravate hepatic lipid accumulation during high-fat feeding [57], but the relatively modest differences in riboflavin, FMN, and FAD in this study are insufficient to infer increased respiratory-chain activity. The cofactor findings are best regarded as an auxiliary redox signal, supported by evidence that glutathione availability and mitochondria–endoplasmic reticulum communication can influence hepatic lipid accumulation and insulin resistance [58,59].

The paired design allowed transcript and metabolite abundance to be compared within the same eight liver samples. Positive nominal correlations included *Gclc* with choline glycerophosphate, *Me1* with LPC(18:0) and LPC(18:1), *Dbi* with LPC(18:0), and *Gpcpd1* with allopregnanolone; *Gclc* was negatively associated with FAD and Oct-5-Enedioylcarnitine. These relationships fit the broader pattern of coordinated differences in lipid handling, phospholipid metabolism, steroid-related metabolism, and redox features. They should not, however, be interpreted as direct regulation, biochemical direction, or substrate–product relationships. No multiple-testing correction was applied to the 49 displayed correlations, and only eight animals were included, with four per group. The selected features were derived from differential analyses and pathway-enrichment results, and both omics datasets partly separated by treatment. The pooled correlations may therefore primarily reflect between-group shifts in transcript and metabolite abundance. High pooled coefficients do not establish associations within either group or associations that persist after accounting for group membership. RT-qPCR corroborated selected transcript-level differences but did not independently validate the gene–metabolite correlations. Multi-omics integration can identify candidate patterns of covariation [60,61], but the present analysis does not establish causal links between the molecular differences and the phenotypic outcomes.

This study is strengthened by its post-obesity intervention design, continued HFD exposure during BM1259 administration, and paired transcriptomic and metabolomic profiling of the same liver samples. Several limitations should be considered. Metabolite annotations from the untargeted platform require targeted confirmation, and the transcriptomic differences were not assessed at the protein, enzyme-activity, or metabolic-flux levels; therefore, the pathways linking BM1259 administration to the hepatic response remain unresolved. Food intake was recorded in only two cages per group, limiting the assessment of differences in energy intake. Similar cage-level means cannot establish equivalent caloric intake or exclude an intake-related contribution to the observed phenotypic differences. Energy expenditure and fecal energy loss were not measured, so the contributions of energy intake, absorption, and expenditure to the lower body weight and hepatic lipid burden remain unresolved. BM1259 persistence, microbiota or intestinal-barrier changes, and metabolites of bacterial origin were also not assessed; accordingly, the hepatic transcriptional and metabolic profiles should be interpreted as responses associated with administration of the bacterial preparation rather than evidence of a defined gut-mediated mechanism. Finally, the use of male mice under a single diet, dose, and intervention duration limits the generalizability of the findings. Within these boundaries, BM1259 warrants further investigation as a candidate microbial intervention in this male post-obesity mouse model; efficacy in other experimental settings and its safety for oral use remain to be established.

## 5. Conclusions

In male C57BL/6J mice with established diet-induced obesity, body weight was significantly lower in the HFD + BM1259 group at the intervention endpoint. BM1259 administration was also associated with lower adipose-tissue burden and reduced hepatic lipid accumulation, as supported by hepatic triglyceride content and Oil Red O morphometry. Serum ALT, LDL-C, and fasting blood glucose were also lower, whereas the reduction in ipGTT AUC did not reach statistical significance, indicating that the glycemic response was partial. These phenotypic differences were accompanied by reduced expression levels of certain liver transcripts associated with lipid uptake and synthesis, which were also validated at the mRNA level. Additionally, changes were observed in the metabolism of choline–glycerophospholipids, steroids, and sphingolipids. Therefore, BM1259 warrants further investigation as a candidate microbial intervention.

## Figures and Tables

**Figure 1:**
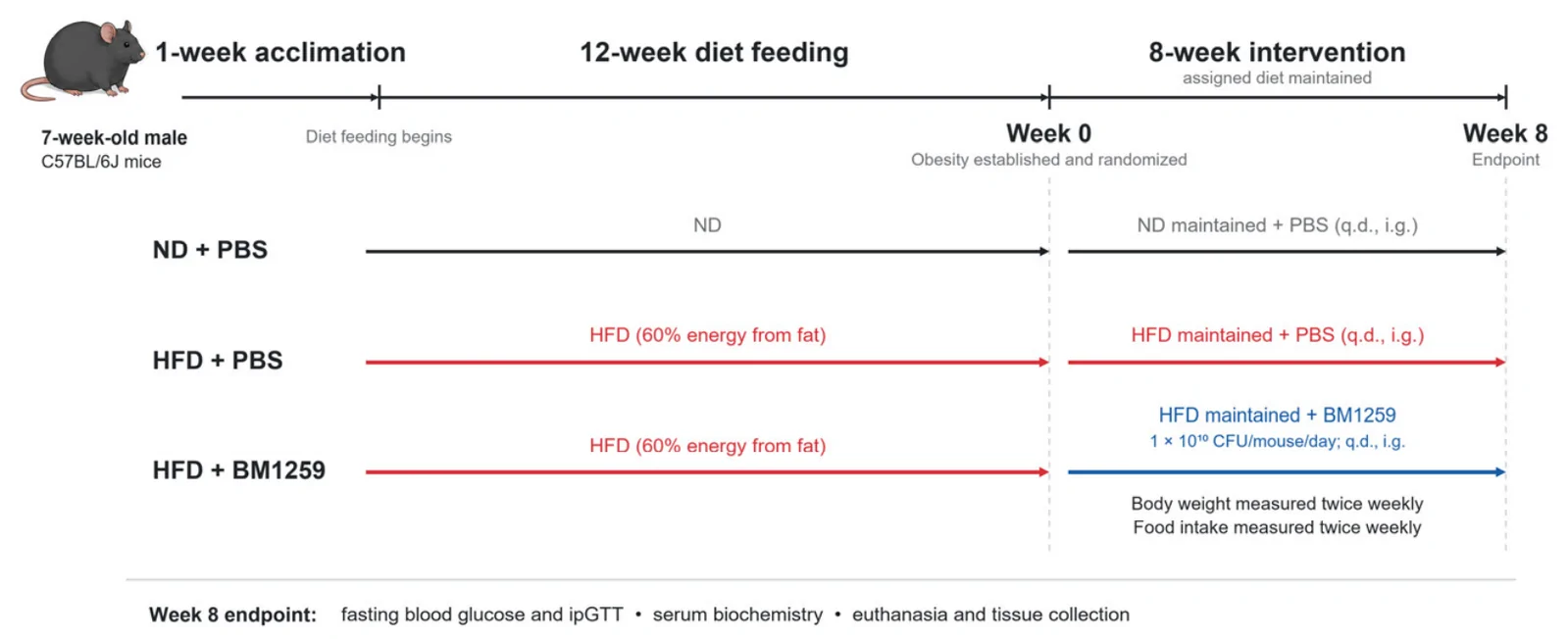
Experimental design. Male C57BL/6J mice were fed a normal diet (ND) or high-fat diet (HFD) for 12 weeks. After diet-induced obesity had been established, eligible HFD-fed mice were assigned to the HFD + PBS or HFD + BM1259 group. The mice remained on their assigned diets for a further 8 weeks, during which BM1259 was administered once daily by intragastric gavage at 1 × 10^10^ CFU/mouse/day. The ND and HFD control groups received PBS.

**Figure 2:**
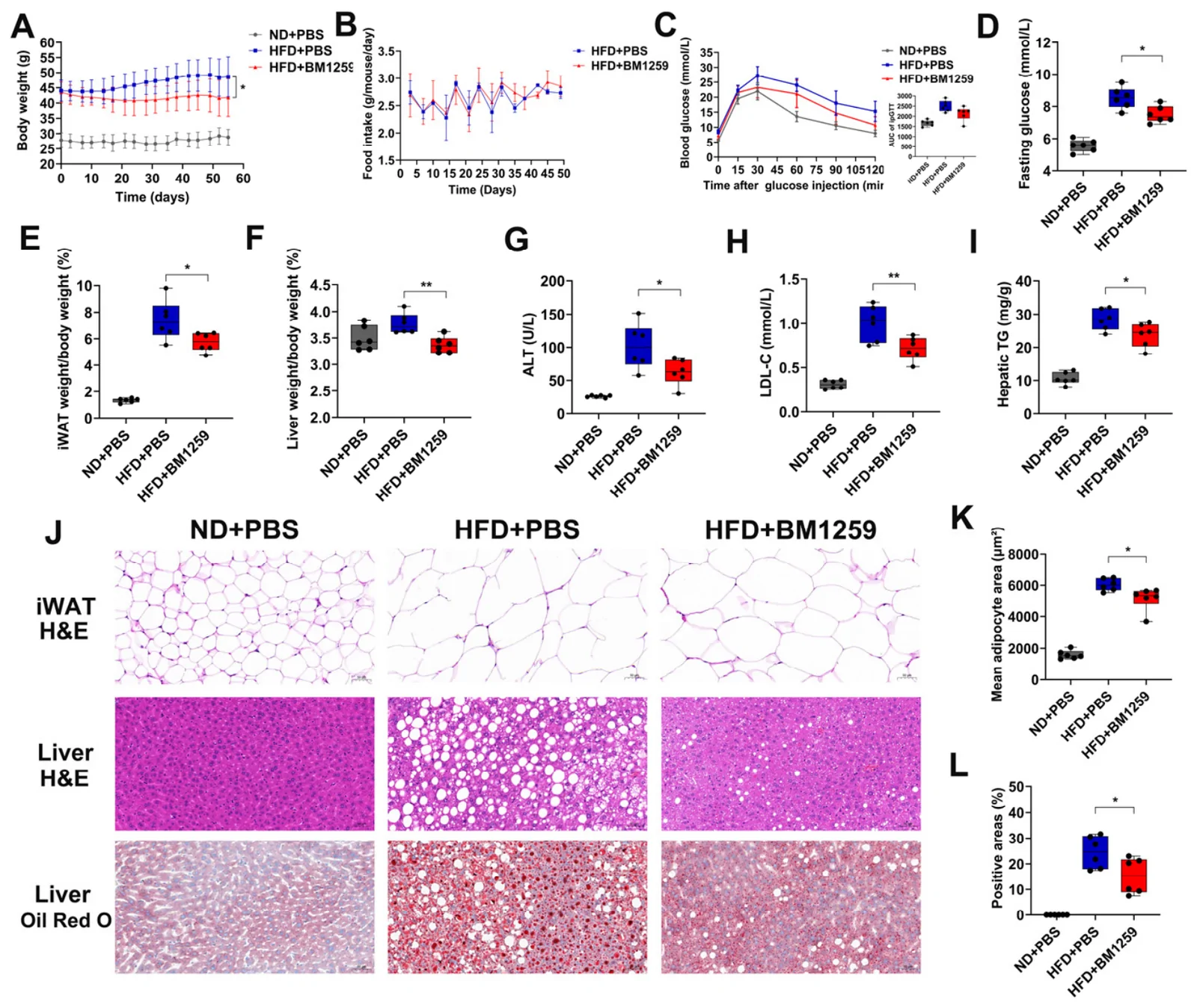
Obesity-related metabolic and hepatic phenotypes following BM1259 administration. (**A**) Body weight during the 8-week intervention. (**B**) Cage-level food intake during the intervention. (**C**) Blood glucose concentrations during the intraperitoneal glucose tolerance test (ipGTT); the inset shows the corresponding area under the curve (AUC). (**D**) Fasting blood glucose at the end of the intervention. (**E**) Inguinal white adipose tissue (iWAT) weight normalized to body weight. (**F**) Liver weight normalized to body weight. (**G**) Serum alanine aminotransferase (ALT). (**H**) Serum low-density lipoprotein cholesterol (LDL-C). (**I**) Hepatic triglyceride (TG) content. (**J**) Representative hematoxylin and eosin (H&E)-stained sections of iWAT and liver and Oil Red O-stained liver sections. (**K**) Mean adipocyte cross-sectional area in iWAT. (**L**) Hepatic Oil Red O-positive area. Scale bars, 50 μm. Data in panels (**A**,**B**), and the main plot of panel (**C**) are presented as the mean ± SD. In the inset of panel (**C**) and panels (**D**–**I**,**K**,**L**), points represent individual mice, boxes indicate the median and interquartile range, and whiskers indicate the minimum and maximum. Body weight was assessed in eight mice per group, and food intake was measured in two cages per group. The ipGTT, fasting blood glucose, relative tissue weights, serum biochemical measurements, hepatic TG content, mean adipocyte area, and hepatic Oil Red O-positive area were assessed in six mice per group. * *p* < 0.05 and ** *p* < 0.01.

**Figure 3:**
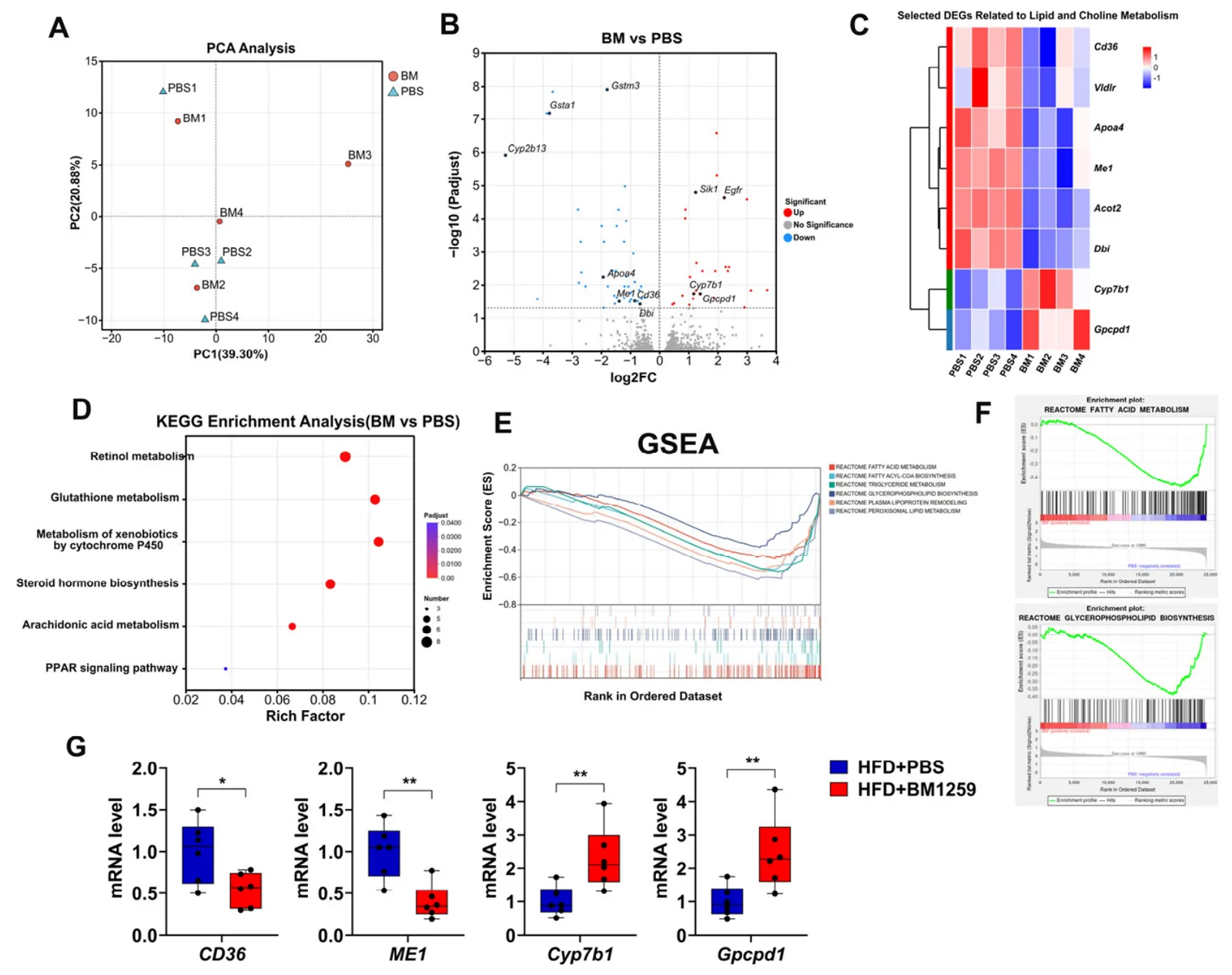
Hepatic transcriptomic profiles of HFD-fed mice following BM1259 administration. (**A**) Principal component analysis of hepatic gene-expression profiles. (**B**) Volcano plot comparing the HFD + BM1259 and HFD + PBS groups. Red, blue, and gray dots denote upregulated, downregulated, and non-differentially expressed genes, respectively. (**C**) Row-standardized heatmap of selected differentially expressed genes related to lipid and choline metabolism. Red and blue denote relatively high and low expression, respectively. (**D**) Kyoto Encyclopedia of Genes and Genomes (KEGG) pathway enrichment analysis of the differentially expressed genes. The x-axis indicates the rich factor; dot size represents the number of mapped genes, and dot color represents the adjusted *p* value. (**E**) Combined gene set enrichment analysis (GSEA) profiles of six selected Reactome gene sets related to hepatic lipid metabolism. (**F**) Individual enrichment plots for the Reactome fatty acid metabolism and glycerophospholipid biosynthesis gene sets. (**G**) RT-qPCR analysis of hepatic mRNA abundance. Negative normalized enrichment scores indicate enrichment toward the HFD + PBS group. GSEA was performed using the complete ranked gene list. Panels (**A**–**F**) were based on four biologically independent mice per group. RT-qPCR analysis in panel (**G**) included six mice per group. * *p* < 0.05 and ** *p* < 0.01.

**Figure 4:**
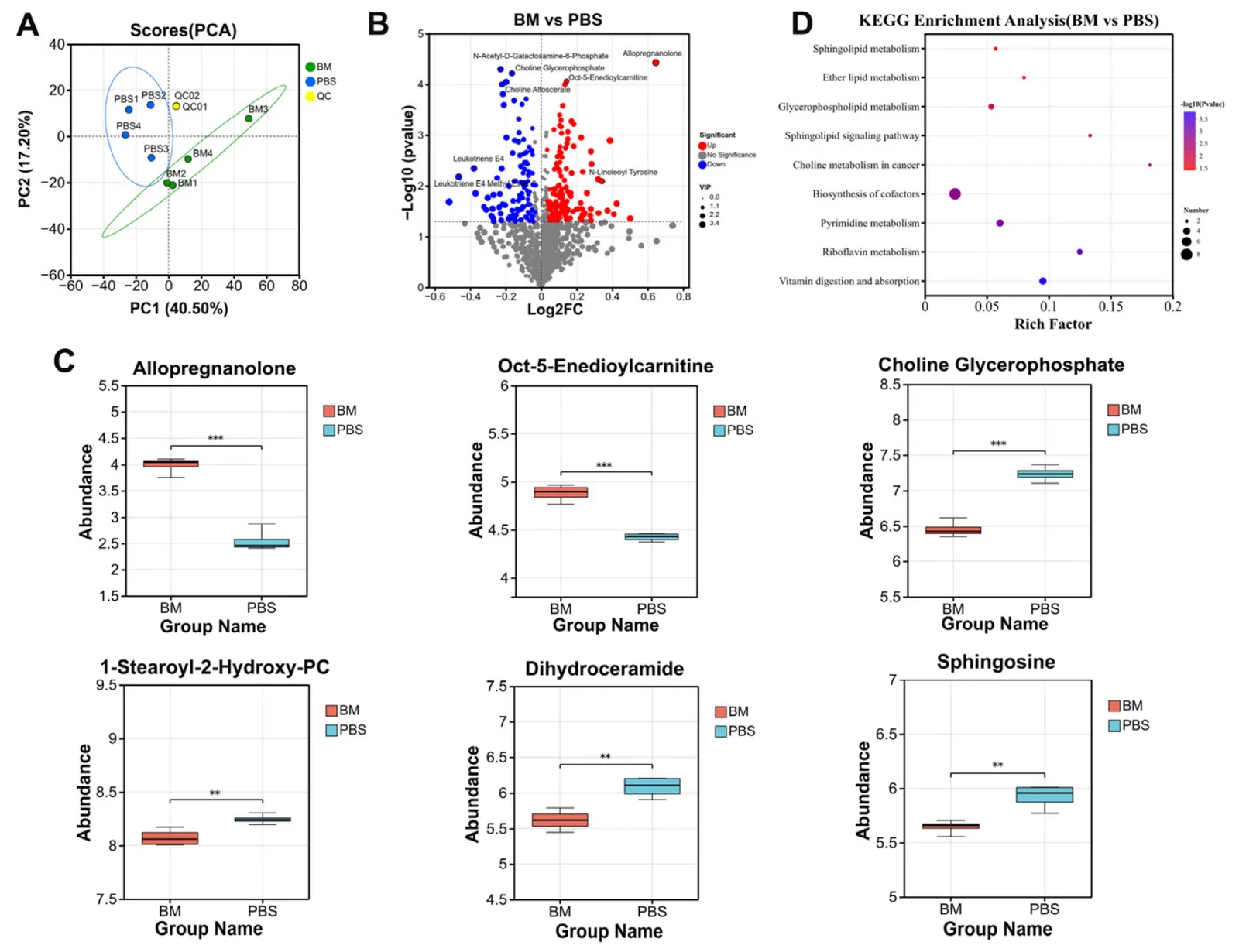
Hepatic metabolomic profiles of HFD-fed mice following BM1259 administration. (**A**) Principal component analysis of hepatic metabolomic profiles. Pooled quality-control samples were included to monitor analytical stability. (**B**) Volcano plot comparing the HFD + BM1259 and HFD + PBS groups. Red, blue, and gray dots denote increased, decreased, and non-differential metabolites, respectively; dot size represents the variable importance in projection (VIP) score. (**C**) Relative abundances of six representative candidate differential metabolites. Boxes indicate the interquartile range and median, and whiskers indicate the range. (**D**) Kyoto Encyclopedia of Genes and Genomes (KEGG) pathway enrichment analysis of the candidate differential metabolites. The x-axis indicates the rich factor; dot size represents the number of mapped metabolites, and dot color represents −log_10_ of the unadjusted *p* value. Candidate differential metabolites were defined by VIP > 1 and a two-sided Student’s *t*-test *p* value < 0.05. ** *p* < 0.01 and *** *p* < 0.001. Data were obtained from four biologically independent mice per group.

**Figure 5:**
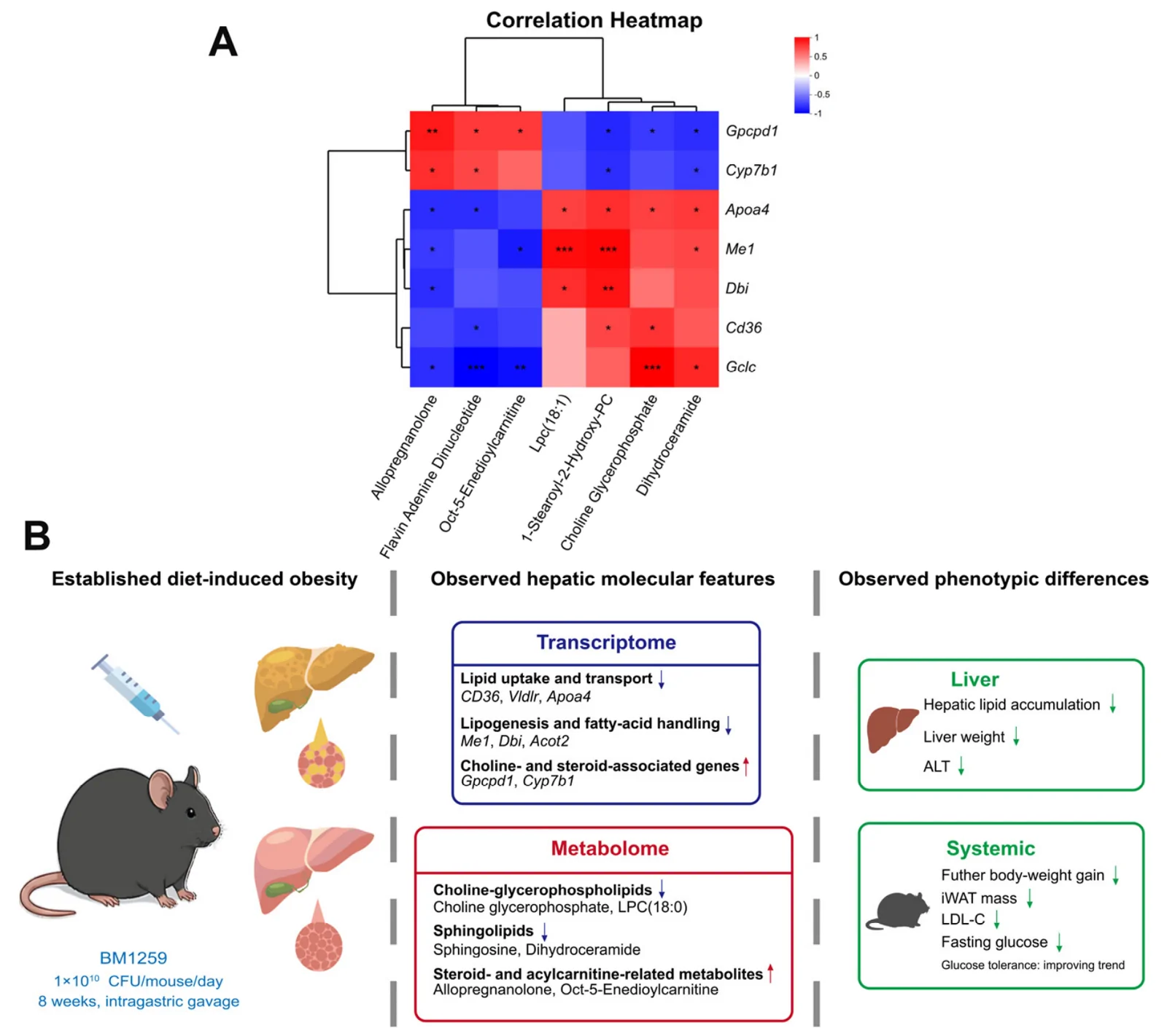
Integrated hepatic multi-omics analysis and descriptive summary of BM1259-associated changes. (**A**) Hierarchically clustered heatmap of Spearman correlations between selected hepatic genes and metabolites across eight matched liver samples. Red and blue denote positive and negative correlations, respectively, and color intensity represents the absolute magnitude of Spearman’s ρ. Asterisks indicate unadjusted two-sided *p* values: * *p* < 0.05, ** *p* < 0.01, and *** *p* < 0.001. (**B**) Descriptive summary of the phenotypic and hepatic molecular changes associated with BM1259 administration. Upward and downward arrows denote increases and decreases, respectively. The integrated analysis included eight matched liver samples, with four mice per group.

## Data Availability

The datasets used in this study are available from the corresponding author upon reasonable request.

## Supplementary Materials

The following supporting information can be downloaded at: https://www.mdpi.com/article/10.3390/nu18213687/s1, Figure S1: Effects of BM1259 on epididymal adipose tissue and additional serum biochemical parameters in mice with established diet-induced obesity; Figure S2: Expression heatmap of all hepatic differentially expressed genes identified between the HFD + PBS and HFD + BM1259 groups; Figure S3: Gene Ontology enrichment analysis of hepatic differentially expressed genes; Figure S4: Hierarchical clustering of hepatic candidate differential metabolites; Table S1: Primer sequences; Table S2: Complete gene-level statistics; Table S3: Complete Gene Ontology, KEGG and reactome GSEA results; Table S4: Complete feature-level statistics of differential metabolites; Table S5: Complete pathway-enrichment results; Table S6: Complete coefficients and nominal *p* values.
