# Supplementary material for: Bacillus megaterium 1259 Limits Hepatic Lipid Accumulation in Mice with Established Diet-Induced Obesity: Paired Hepatic Transcriptomic and Metabolomic Profiling
Source: Nutrients. 2026 Nov 6;18(21):3687. doi: 10.3390/nu18213687 (PMC13765034; doi:10.3390/nu18213687)
Supplement: Supplementary file 1 [file nutrients-18-03687-s001.zip › Figures S1-S4.pdf]

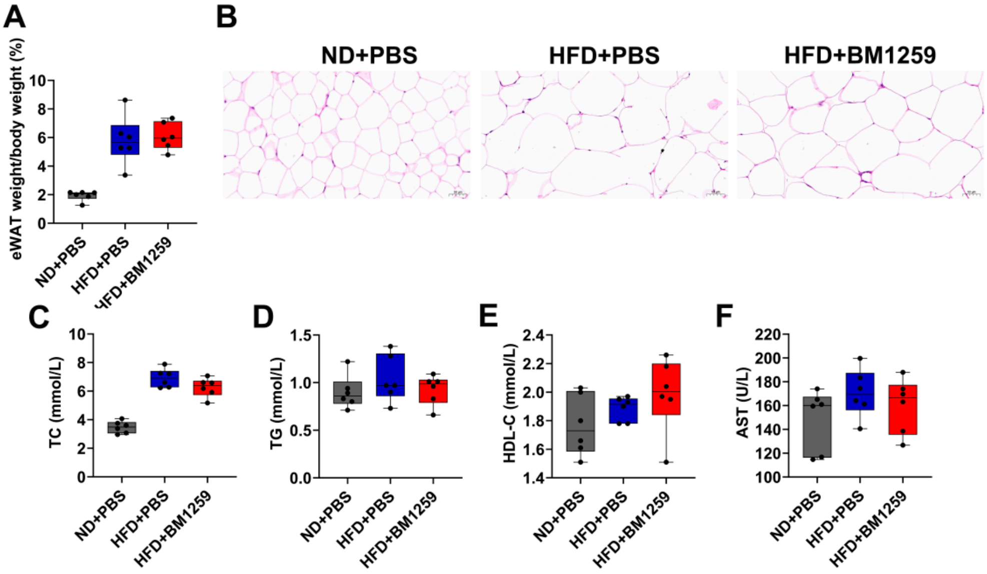

Figure S1. Effects of BM1259 on epididymal adipose tissue and additional serum biochemical parameters in mice with established diet-induced obesity. (A) Epididymal white adipose tissue (eWAT) weight normalized to body weight. (B) Representative hematoxylin and eosin (H&E)-stained sections of eWAT from the ND + PBS, HFD + PBS, and HFD + BM1259 groups. Scale bar, 50 μm. (C–F) Serum total cholesterol (TC) (C), triglycerides (TG) (D), high-density lipoprotein cholesterol (HDL-C) (E), and aspartate aminotransferase (AST) activity (F). Quantitative data are presented as box-and-whisker plots with individual values (n = 6 mice per group). Group differences were evaluated using one-way ANOVA followed by Tukey's multiple-comparisons test.

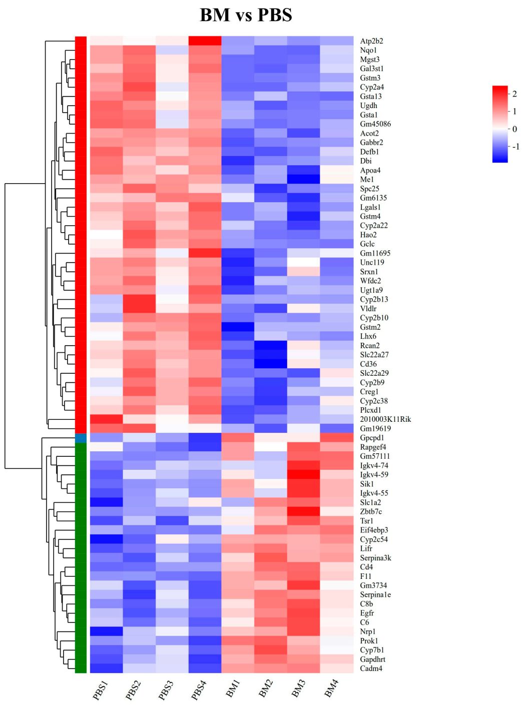

Figure S2. Expression heatmap of all hepatic differentially expressed genes identified between the HFD + PBS and HFD + BM1259 groups. Expression values were standardized by gene and are displayed as row z-scores. Red and blue indicate relatively high and low expression, respectively; n = 4 mice per group.

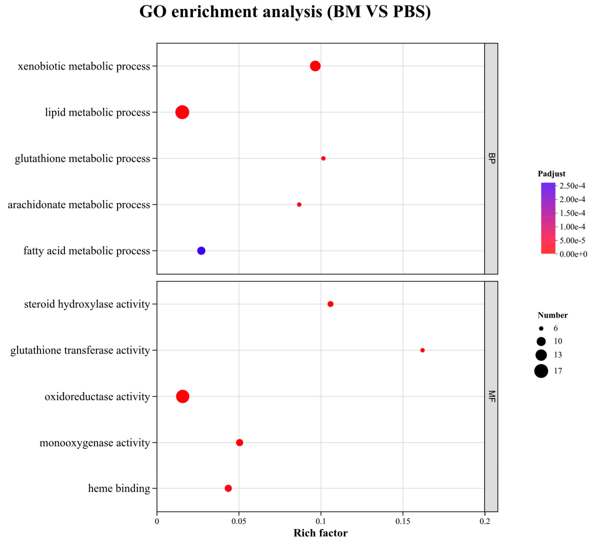

Figure S3. Gene Ontology enrichment analysis of hepatic differentially expressed genes. Dot size represents the number of mapped genes, and dot color represents the adjusted p value. BP, biological process; CC, cellular component; MF, molecular function.

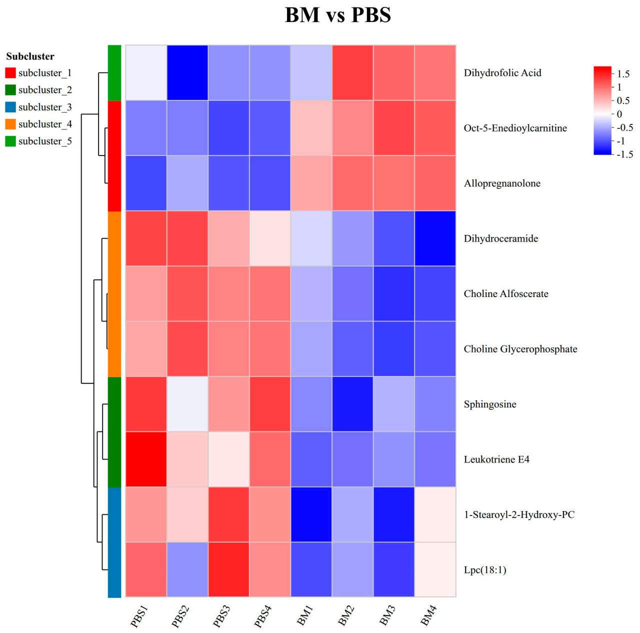

Figure S4. Hierarchical clustering of hepatic candidate differential metabolites. Relative metabolite abundances were standardized across samples. Red and blue indicate relatively high and low abundance, respectively; n = 4 mice per group. Metabolite annotations are putative.
